# The Role of* Nigella sativa* and Its Active Constituents in Learning and Memory

**DOI:** 10.1155/2016/6075679

**Published:** 2016-02-28

**Authors:** Mohamad Khairul Azali Sahak, Nurul Kabir, Ghulam Abbas, Suhaimi Draman, Noor Hashida Hashim, Durriyyah Sharifah Hasan Adli

**Affiliations:** ^1^Division of Biohealth Science, Institute of Biological Sciences, Faculty of Science, University of Malaya, 50603 Kuala Lumpur, Malaysia; ^2^Pharmacology Section, HEJ Research Institute of Chemistry, International Centre for Chemical and Biological Sciences, University of Karachi, Karachi 75270, Pakistan; ^3^Division of Biology, Centre for Foundation Studies in Science, University of Malaya, 50603 Kuala Lumpur, Malaysia

## Abstract

The loss of the ability for learning and memory is a prominent feature of dementia, which affects millions of individuals all over the world, due to either neurodegenerative diseases or brain injury. Although a lot of information is known about the pathology involved, treatment remains elusive at best. The Black Seed of* Nigella sativa* has been historically and religiously used for thousands of years for preventing and treating many different kinds of diseases. This review article looks at* Nigella sativa* and its potential role in facilitating learning and memory. The possible use of this seed's extract or compounds isolated from it, such as thymoquinone, for treating damaged brain neural tissue is discussed. The evidence presented in this paper appears to be supporting the hypothesis that this plant and/or its bioactive constituents can enhance learning and memory in health and disease in animals and humans.

## 1. *Nigella sativa* History and Its Importance

Being an established historical and religion-based remedy for wide ranging health problems,* Nigella sativa* (NS), which belongs to the family Ranunculaceae, is one of the herbal medicines that has been extensively investigated and gaining worldwide recognition [1]. NS is a native dicotyledonous plant to southern Europe, North Africa, and Asia Minor, and being widely cultivated in Pakistan and India, thus, becoming a household traditional medicinal plant in the region [2]. Over the years, immigration helped the plant cultivation to spread extensively throughout Eastern Europe and North America. It is also known as the Black Seed because when the seeds are exposed to air, they turned black [1]. Among the Muslim community, this plant is referred to as* Habbatus Sauda*,* Alhabahat Alsawda*, and* Alkamoun Alaswad* in reference to the colour of its seeds [3]. In some other parts of the world, it is also known as* Shuniz*,* Khodhira*, Black Cumin, or Black Caraway [4].

NS has a long history of folklore usage in different civilizations and has been recognized as a “miracle cure” for its ability to treat various diseases and assist the body in its own natural healing process [1]. In ancient texts and historical documents, NS has been mentioned as a notable healer for a range of ailments. Archeological evidence about the earliest cultivation of NS is scanty but there are studies, which reported that NS seeds have been found in several sites from ancient Egypt, including in the tomb of Tutankhamen. It is also known as a beauty secret since ancient times as Queen Nefertiti, who was praised for her exquisite complexion, was a devoted user of NS oil [5]. The earliest written reference is in the book of Isaiah of the Bible, in which it is referred to as “*Ketzah*” in Hebrew, a spice for bread and cakes [6].

For the Muslim community, the traditional practice of its usage is primarily due to the authentic prophetic statement that NS is a cure for all, except death; that was quoted by a renowned Muslim scholar, Al-Bukhari [7]. Thus, the glorified status of NS among the Muslim community is as* Habbat Albarakah*, with the term “*Albarakah*” signifying its “blessed” status [3]. Besides that, various Muslim scholars also gave ample credit to the healing properties of the NS and, hence, its importance in the “Prophetic Medicine” tradition. The Persian physician and philosopher Ibn Sina, commonly known in the West as Avicenna, had mentioned NS in his famous medical treatise “Canon of Medicine,” which is considered as a hallmark in the history of human medicine and was used as the main medical text until the 17th century in Europe. In his writings, he stated that NS has preventative and restorative features as it stimulates the body's energy and helps in recovery from fatigue or dispiritedness. Ibn Sina also recommended NS as a remedy for fever, common colds, headache, toothache, skin diseases, wounds, fungus, parasites, and worms as well as against bites and stings by poisonous animals [5].

NS has been reported to have many therapeutic properties such as immunopotentiation, bronchodilatation, and being antitumor, antihistaminic, antidiabetic, antihypertensive, anti-inflammatory, antimicrobial, hepatoprotective, and gastroprotective, which are attributed to its quinone constituents in the seeds [8–10]. Identification of the therapeutic features of NS came from researches in various fields starting in the early 1970s [11]. Nonetheless, there are comparatively only a few studies that scientifically support its positive role in treating central nervous system (CNS) related ailments. However, considering its significant antioxidant, anti-inflammatory, and immunomodulatory properties, consuming NS could be one of the promising health strategies to help prevent the oxidative damage to cells, particularly in the brain regions related to memory functions [12]. Thus, this review article looks at NS and its potential role in facilitating learning and memory. The possible use of this seed's extract or compounds isolated from it, such as thymoquinone (TQ), for treating neurodegenerative disease is discussed. The evidence presented in this paper appears to be supporting the hypothesis that this plant and/or its bioactive constituents can enhance learning and memory in health and disease in animals and humans.

## 2. Bioactive Constituents of* Nigella sativa*

**Figure 1:**
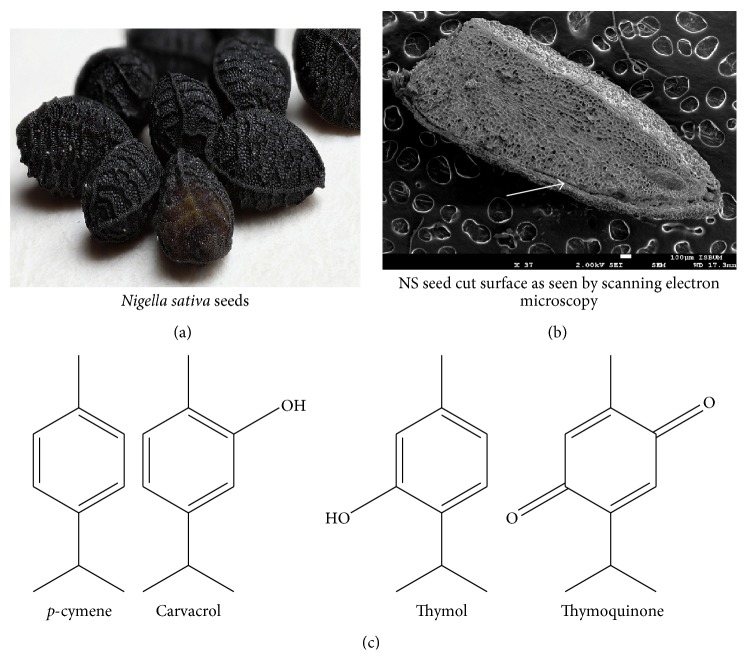
(a) Each* Nigella sativa* (NS) seed shows characteristic corrugations of its surface. (b) NS essential oil resides in vesicles just beneath the black seed coat (as shown by white arrow). (c) NS oil is mainly composed of monoterpenes (having 10 carbon atoms) having phenolic groups that provide the basis for its antioxidant activity.

Literature revealed that from ancient times it has been known that the medicinally significant component of the NS plant is the* Nigella sativa* oil (NS oil) (Figure 1). The efficacy of the NS oil is mostly attributed to its quinone constituents in the NS fixed and essential oil, which is especially endowed with thymoquinone (TQ), a significant bioactive constituent making up 30–48% of the total compounds [13]. Other functional components of the NS oil include* p*-cymene, carvacrol, thymohydroquinone (THQ), dihydrothymoquinone (DHTQ), *α*-thujene, thymol,* t*-anethole, *β*-pinene, *α*-pinene, and *γ*-terpinene.

Among these, TQ has received the most attention and is mostly attributed to the learning and memory enhancing effects of NS. It has been shown to ameliorate diabetes-induced cognitive decline by preventing oxidative stress [14]. TQ has also been reported to restore oxidative balance, mitochondrial dysfunction, and cholinesterase activity caused by A*β* administration to PC 12 cells [15]. It exhibited a neuroprotective effect in hippocampal slices and cultured rat primary neurons treated with A*β* [16, 17]. It is further shown to inhibit apoptosis induced by A*β* in primary cultured cerebellar granule neurons [18]. In addition, TQ and THQ are usually present in the form of glycosidically bound aglycones, which easily cross the blood-brain barrier, hence, possibly related to its neuroprotective effects [19]. TQ has also been shown to inhibit nonenzymatic peroxidation in ox brain phospholipid liposomes with a 10 times higher potency than NS oil [20]. Taken together, TQ appears to be the major neuroprotective constituent present in NS oil.

The other bioactive compounds, that is, thymol and carvacrol, also attenuated A*β*- and scopolamine-induced cognitive impairments in rats [21]. Both of the aforesaid bioactive compounds along with *γ*-terpinene and* p*-cymene are shown to inhibit the acetylcholinesterase activity while *γ*-terpinene alone is found to be a good inhibitor of lipid peroxidation [19, 22]. Notably, a nutraceutical containing thymol and* p*-cymene has been patented for cognitive enhancement properties [23]. Therefore, it appears that the cholinergic modulation properties of NS may be mediated by constituents other than TQ.

Flavonoids are present in NS seeds and have been widely studied [24–26]. Emerging evidence suggests that flavonoids are able to induce improvements in memory, learning, and cognition. Flavonoids have been shown to modulate critical neuronal signaling pathways involved in processes of memory and, therefore, are likely to affect synaptic plasticity and long-term potentiation (LTP) mechanisms, which is widely considered as a mechanism for memory [27]. Briefly, flavonoid-induced improvements in behaviour have been associated with specific changes in protein expression in the hippocampus. Hippocampal elevation of NR2B-containing N-methyl-D-aspartate (NMDA) receptor at synaptic sites is correlated with the levels of the adhesion molecule of polysialylated form of the neural adhesion molecule (PSA-NCAM) in the dentate gyrus of the hippocampus, with both proteins linked to efficient and persistent LTP and spatial learning [28].

## 3. Effects of* Nigella sativa* on Learning and Memory

Learning and memory are the most important executive functions performed by the human brain, the loss of which is a prominent feature in dementia. Dementia can be caused by aging, physical and/or chemical injuries, or neurodegenerative diseases, which in most cases would affect the quality of learning and memory of the concerned individuals. The latter include health problems such as Alzheimer's disease (AD) or Parkinson's disease (PD), which are characterized by the accumulation of protein aggregates on the surface or inside the neurons. Disturbances, which cause oxidative stress and elevated cortisol levels, can lead to neurodegeneration that may subsequently induce a fall in cognitive ability. Any chemical, natural, or synthetic substances that enhances executive functions of the brain is of immense clinical significance.

In comparison to studies involving other plant materials, established reports on the effects of NS seeds and/or its constituents on the CNS and on behavioural actions are few, most of which focused on the spatial memory [29]. Spatial memory involves memory for spatial information by which the brain functions in recognizing, codifying, storing, and recovering information about objects or routes. It has working memory and reference memory components and normally associated with exploratory behaviour and curiosity, which represent the need to acquire information when facing new environments [30].

It is well known that cholinergic neurons are degenerated in AD and, notably, acetylcholine (ACh) as a neurotransmitter plays a role in facilitating learning and memory, and, therefore, its decreased release will result in memory impairment. Hence, elevation of ACh via the inhibition of its degradation by acetylcholinesterase (AChE) is a currently used strategy for its management. Pharmacological studies demonstrated that NS is involved in AChE inhibition activity, the principal enzyme involved in the hydrolysis of Ach, thus, retaining its effects in the encoding of new memories.

### 3.1. Studies on Animals

The involvement of the central cholinergic enhancement (via AChE inhibition) is reflected from the alleviating effect by NS hydroalcoholic extract against scopolamine-induced amnesia [31]. The mnemonic effect, cholinergic modulation, and oxidative stress mitigation were attributed to the oil present in NS [29]. A study has also reported that extract of NS could prevent scopolamine-induced deficit memory in rats, as the animals showed better performance in passive avoidance test and decreased AChE activity in the hippocampus and cortex tissue of the brain [32]. Following scopolamine administration, NS treated group decreased the AChE activity and oxidative stress of the brain cortex tissues in rats, as evidenced by significant decrease in total sulfhydryl (SH) and increase in malondialdehyde (MDA) and thiol concentrations [33]. Worth noting is the fact that NS oil tended to mimic the effects of donepezil, an AChE inhibitor, which is known to have positive effects by decreasing MDA and brain tumor necrosis factor-alpha (TNF-*α*) content as well as increasing glutathione brain contents. Oral pretreatment of NS oil could significantly reverse the amnesic effect of scopolamine-induced deficit of spatial and nonspatial working memory impairment in the T-maze alternation task and object recognition test, respectively [34].

Induced neurotoxicity by A*β*-peptide, a protein type which is commonly accumulated in AD, could be protected by NS oil and its aqueous fraction via antioxidant effect in rat primary cerebellar neurons [35]. Its oil further showed beneficial effect on memory in animal model of chronic hypoperfusion without altering the hippocampal plasticity and preserving the ultrastructural constituents [36–38].

Some inferences are also drawn from works done on diabetes, which is characterized by hyperglycemia, and reported to be associated with cognitive decline. A study conducted by Khan and colleagues has shown that TQ, the active principle of NS, has neuroprotective properties on cognitive impairment and related dementias [39]. Rats pretreated with 3 mg/kg body weight of TQ for 15 days after streptozotocin- (STZ-) induced cognitive impairment have been found to significantly decrease latency and path length in the Morris Water Maze (MWM) behaviour test and restored antioxidant enzymes viz. glutathione reductase, glutathione peroxidase, superoxide dismutase, and catalase. NS extract has also been shown to ameliorate spatial memory disturbances linked with diabetes in rodents as shown through the use of passive avoidance and Y-maze tests, indicated by improved initial latency, step-through latency, and alternation behaviour [40]. Importantly, in diabetic rats, the aqueous extract of NS is shown to have adaptogenic effect via normalizing the hypothalamus-pituitary-adrenal (HPA) gland axis and oxidative stress [41, 42]. These actions probably underlie the aforementioned protective effect of NS in diabetic rats.

Our research team has also reported the possible beneficial effects of NS oil administration on the spatial memory performance (SMP) of male adult rats using the radial arm maze (RAM) apparatus, one of the standard apparatuses used in behavioural-based research to assess spatial memory [43]. From the finding, it is reasonable to suggest that treatment with NS oil could enhance the learning ability and memory of the rats, especially the working memory.

Eysenck and Calvo suggested that anxiety could also partly impair memory performances, depending on certain circumstances [44]. For instance, anxious individuals have less attentional capacity for task performance and, thus, do not perform as well as nonanxious individuals on tasks that make substantial demands on working memory [45]. NS has been also demonstrated to produce antianxiety effect in different tests which used behavioural models for exploration-induced anxiety. One study confirmed this hypothesis; NS daily treatment for four weeks exhibited increase in the open field activity and produced antianxiety behaviour when tested in elevated plus maze. Treatment with NS also increased levels of serotonin/5-hydroxytryptamine (5-HT) and decreased the levels of hydroxyindoleacetic acid (5HIAA) in the brain, both inducing the coordination of behaviour including reducing anxiety via the production of serotonin [46].

Epilepsy, a neuro-related disease characterized by seizures, can also lead to poor cognitive functions. In the pentylenetetrazole- (PTZ-) induced epileptic model, the NS hydroalcoholic extract was reported to be beneficial by preventing the learning and memory decline [47]. In addition, glycation, the nonenzymatic reaction between sugar and protein, is the phenomenon that is long known to underlie several aging linked physiological alterations. It is suggested that NS may affect the glycation process, although the phenomenon remains elusive, and hence, is worth investigating.

Thyroxine plays an important role in growth, development, and function of the brain. In neonatal animals, hypothyroidism linked with learning and memory impairments could be reversed by hydroalcoholic extract of NS, which is attributed to its antioxidant effects. Comparable with vitamin C, NS treatment reduced the time latency, increased the time spent in target quadrant in MWM test, and significantly increased the time latency for entering the dark compartment in passive avoidance test [48, 49]. This data reflects the neuronal growth promoting effect of NS and should be evaluated in CNS retardation studies.

### 3.2. Studies on Humans

Literature reveals that NS possesses mnemonic/nootropic properties. In elderly humans, its commercially available capsule (500 mg for 9 weeks) was also shown to enhance the executive functions in various memory related tests such as logical memory, digit span, letter cancellation, Rey-Osterrieth complex figure, trail making, and stroop tests [50]. The effects of NS on mood, anxiety, and cognition have also been investigated in human subjects [51]. Volunteers were assessed for cognition with modified California verbal learning test-II (CVLT-II), mood with Bond-Lader scale, and anxiety with State-Trait Anxiety Inventory (STAI). Four weeks daily consumption of one NS capsule of 500 mg as a nutritional supplement stabilized mood, decreased anxiety, and improved memory.

## 4. Conclusions

The neuroprotection plus cholinergic modulation by NS provides a good example of the emerging multitarget approach towards treating complex ailments such as AD. Though the literature has revealed several reports addressing the effects of NS and its bioactive constituents on learning and memory, its mechanism of action still remains elusive. Long-term potentiation (LTP), amyloid precursor protein cleaving enzymes, glutamatergic system, GABAergic neurotransmission, mitochondrial membrane, and enzymes are other important modulators of learning and memory, which need to be investigated in the context of the aforementioned mnemonic/nootropic effects of NS.

Taken together, these mentioned reports in this review are strongly suggestive of the neuroprotective potential of NS and/or its bioactive constituents in animals and humans. It appears that enough data has been accumulated to support NS as a potential candidate for a drug discovery programme against neurodegeneration related diseases and brain injury affecting learning and memory.
